# Supplementary material for: Charge order driven by multiple-Q spin fluctuations in heavily electron-doped iron selenide superconductors
Source: Nat Commun. 2023 Apr 11;14:2023. doi: 10.1038/s41467-023-37792-3 (PMC10090174; doi:10.1038/s41467-023-37792-3)
Supplement: Supplementary file 1 — Supplementary Information [file 41467_2023_37792_MOESM1_ESM.pdf]

Supplementary Materials for

"Charge order driven by multiple-**Q** spin fluctuations in heavily electron-doped iron selenide superconductors"

Ziyuan Chen[1†], Dong Li[2,3†], Zouyouwei Lu[2,3], Yue Liu[2,3], Jiakang Zhang[1], Yuanji Li[1], Ruotong Yin[1], Mingzhe Li[1], Tong Zhang[4,5,6], Xiaoli Dong[2,3,7], Ya-Jun Yan[1*], Dong-Lai Feng[1,5,6*]

[1] *School of emerging Technology and Department of Physics, University of Science and Technology of China, Hefei 230026, China*

[2] *Beijing National Laboratory for Condensed Matter Physics, Institute of Physics, Chinese Academy of Sciences, Beijing 100190, China*

[3] *School of Physical Sciences, University of Chinese Academy of Sciences, Beijing 100049, China*

[4] *Department of Physics, State Key Laboratory of Surface Physics and Advanced Material Laboratory, Fudan University, Shanghai 200438, China*

[5] *Collaborative Innovation Center of Advanced Microstructures, Nanjing, 210093, China*

[6] *Shanghai Research Center for Quantum Sciences, Shanghai, 201315, China*

[7] *Songshan Lake Materials Laboratory, Dongguan, Guangdong 523808, China*

1. Structural and superconducting properties of $(Li_{0.84}Fe_{0.16}OH)Fe_{1-x}Se$ films

Figure S1 shows the structural and superconducting properties of four $(Li_{0.84}Fe_{0.16}OH)Fe_{1-x}Se$ films (labeled as #1-#4) that were used in our STM study. Crystal structures of these films were characterized by x-ray diffraction (XRD). Figure S1a shows the measurements of θ-2θ scan, only the (00*l*) peaks of $(Li_{0.84}Fe_{0.16}OH)Fe_{1-x}Se$ films and $LaAlO_3$ substrates are obvious, confirming a single preferred (001) orientation of the films. As shown in Fig. S1b, the out-of-plane crystal mosaic of the films is very small for superconducting samples, in the range of 0.08° to 0.18° in terms of the full width at half maximum (FWHM) of the rocking curves of the (006) Bragg reflection peaks, while it is a little larger for the non-superconducting #4 sample.

Superconductivity of these films was checked by the magnetic susceptibility measurements. Samples #1-#3 exhibit obvious diamagnetic signals in the zero-field-cooled (ZFC) magnetic susceptibility curves under $H = 1$ Oe, as shown in Fig. S1c, and the corresponding superconducting transition temperature (T_c) is determined as 42 K, 28 K and 8 K for #1-#3 films, respectively. Sample #4 is non-superconducting down to 1.8 K as confirmed by the absence of diamagnetic signals. As seen in Fig. S1d, T_c increases with the lattice expansion along *c*-axis (here the *c*-axis lattice parameters of samples #1-#4 were calculated from the XRD data shown in Fig. S1a), which is consistent with previous reports[1,2].

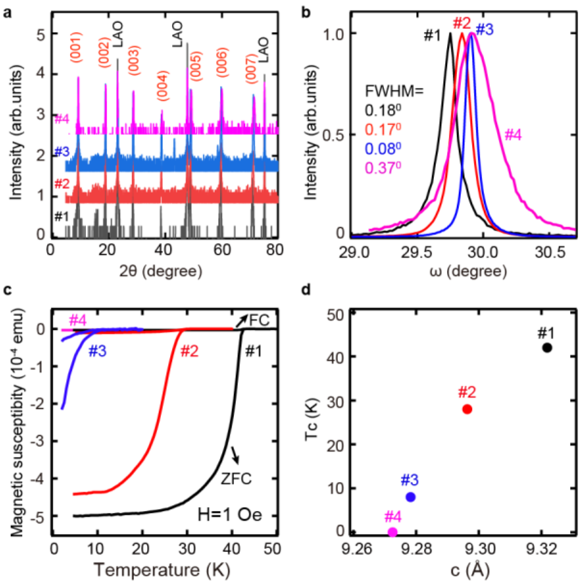

Fig. S1 Structural and superconducting properties of $(Li_{0.84}Fe_{0.16}OH)Fe_{1-x}Se$ films. a XRD patterns of (00*l*) peaks for four $(Li_{0.84}Fe_{0.16}OH)Fe_{1-x}Se$ films grown on $LaAlO_3$ substrates. **b** Rocking curves of the (006) Bragg reflection peaks and corresponding FWHM values. **c** Temperature dependence of the diamagnetic susceptibilities. **d** T_c as a function of the calculated c-axis lattice parameter (c) for these four films, which shows a positive correlation behavior.

2. Two types of Fe-site defects and their influence to superconductivity

In our STM study, we find that the dumbbell-shaped Fe-site defects can be roughly classified into two types according to their impurity potentials. The type-I Fe-site defects are most commonly observed and probably Fe vacancies (V_{Fe}), which process strong impurity scattering potential; They strongly suppress the superconductivity and induce sharp in-gap Yu-Shiba-Rusinov (YSR) states (blue curve in Fig. S2c), and obvious QPI patterns appears around them (Fig. S2b). The type-II Fe-site defects are enclosed by the yellow dashed circles in Fig. S2a,b, and are possibly substitutional defects. They possess much weaker impurity scattering potentials and mainly affect the superconducting state at gap edges (red curve in Fig. S2c), the surrounding QPI signals are very weak compared with the type-I defects.

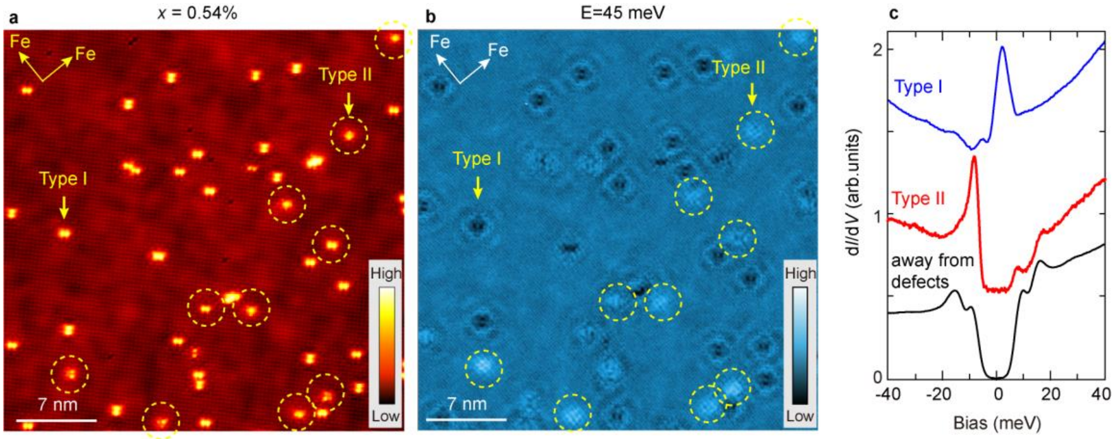

Fig. S2 Two types of Fe-site defects and their influence to superconductivity. a Atomically resolved topographic image of FeSe layer. Type-II Fe-site defects are enclosed by the yellow dashed circles, while the others are the type-I defects. **b** d*I*/d*V* map at E=45 meV. **c** Typical d*I*/d*V* spectra collected on the two

types of Fe-site defects and at the locations far away from the defects.

3. Spatial homogeneity of the d*I*/d*V* spectra on various $Fe_{1-x}Se$ surfaces

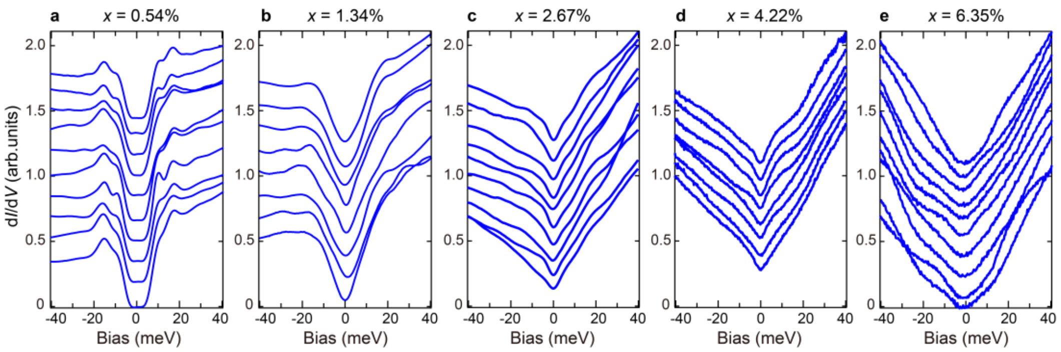

Fig. S3 Typical d*I*/d*V* spectra within ±40 meV measured at different spatial locations of various defect-free $Fe_{1-x}Se$ regions. The spectra are shifted vertically for clarity and they are basically homogeneous in these $Fe_{1-x}Se$ regions.

4. Additional datasets for optimally superconducting $Fe_{1-x}Se$ region with $x = 0.54\%$

Figure S4 shows additional datasets of d*I*/d*V* maps taken in the $Fe_{1-x}Se$ (x = 0.54%) region shown in Fig. 1a. The raw FFT images, smoothed FFT images and the iFFT images with FFT intensities around $\mathbf{q}_{Fe}$ and $\mathbf{q}_{2Fe}$ considered are listed as well. We find that both types of Fe-site defects suppress superconductivity and pin the checkerboard pattern. For d*I*/d*V* maps shown in the first column of Fig. S4, it is obvious that the checkerboard pattern is pinned at type-II Fe-site defects; while it is not so certain for the type-I defects due to the influence of surrounding strong LDOS oscillations which may overwhelm the weak checkerboard pattern, the iFFT images after filtering out QPI signals indeed show pinned checkerboard patterns at both the type-I and type-II defects (see the last column in Fig. S4), but not at Se vacancies or defect-free FeSe areas.

In addition, we have studied the spatial distribution of YSR states induced by the most commonly observed type-I Fe-site defects by using high-resolution STM/STS. As show in Fig. S5 (reproduced from another manuscript of ours that is under review), two pairs of YSR states located at ±3.1 meV and ±5.0 meV are observed. They display long-range LDOS oscillations with a wave vector of $2\mathbf{k}_F$ (period ≈ 1.9 nm), and persists up to ~10 nm away from the defect. The oscillation phase shift is different for different YSR states, which is clearly distinguished from the static checkerboard pattern with a period of $2a_{Fe}$ ≈ 0.54 nm. Moreover, at ultralow temperature of 20 mK, these YSR states induced LDOS modulation is only significant at the energies of YSR peaks. At 4.2 K, the YSR peaks are broader, but their spatial distribution does not change. Therefore, the YSR state behaves differently from the observed checkerboard charge order in both spatial distribution and energy scale, thus excluding it as the possible origin of the checkerboard order.

Figure S6 shows the influence of Se vacancies on LDOS. Se vacancies have no influence on superconductivity of $Fe_{1-x}Se$ plane, neither do they pin the checkerboard pattern.

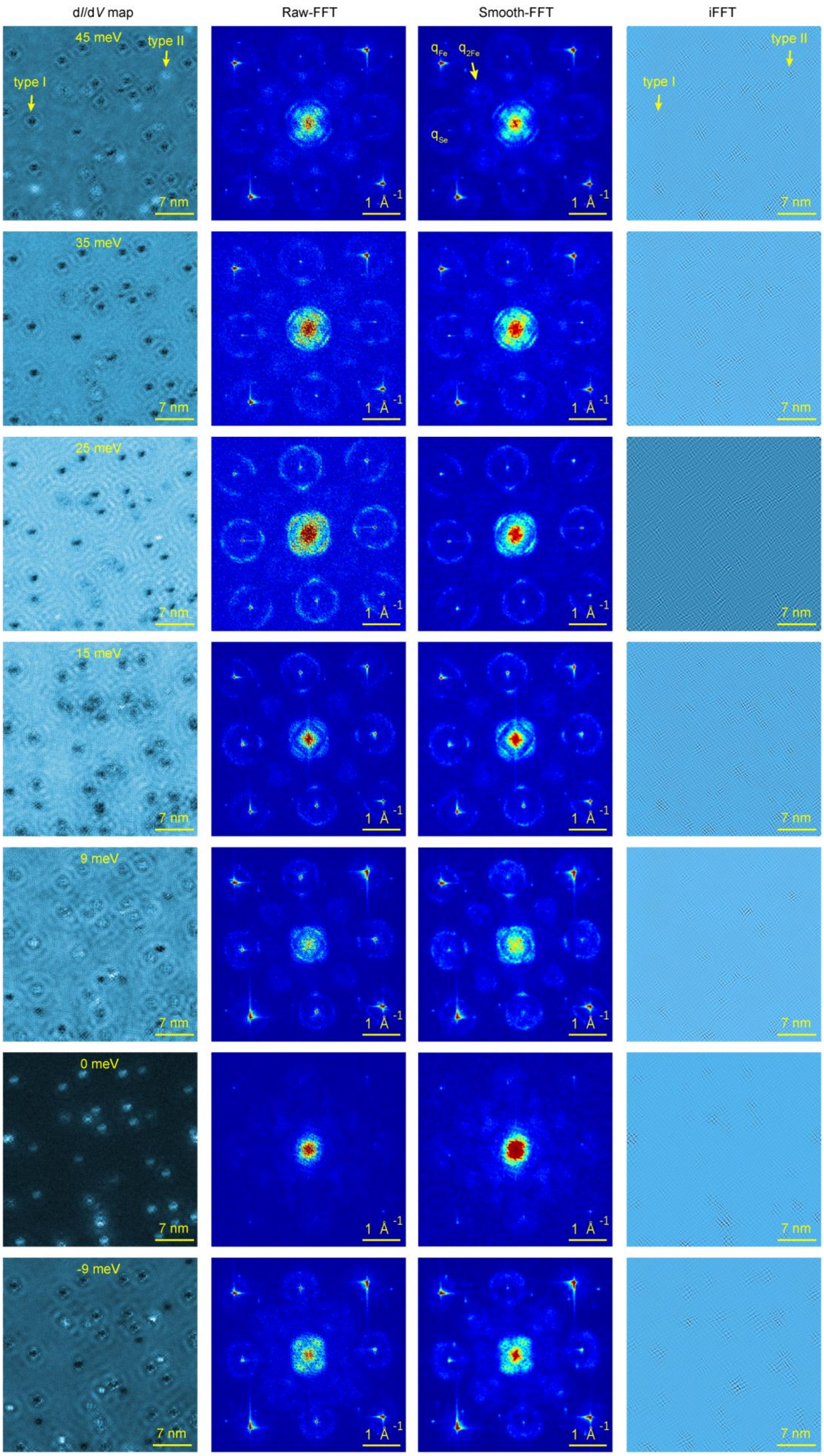

dI/dV map
Raw-FFT
Smooth-FFT
iFFT
45 meV
type II
type I
q_{Fe}
q_{2Fe}
q_{Se}
35 meV
25 meV
15 meV
9 meV
0 meV
-9 meV
7 nm
1 Å^{-1}

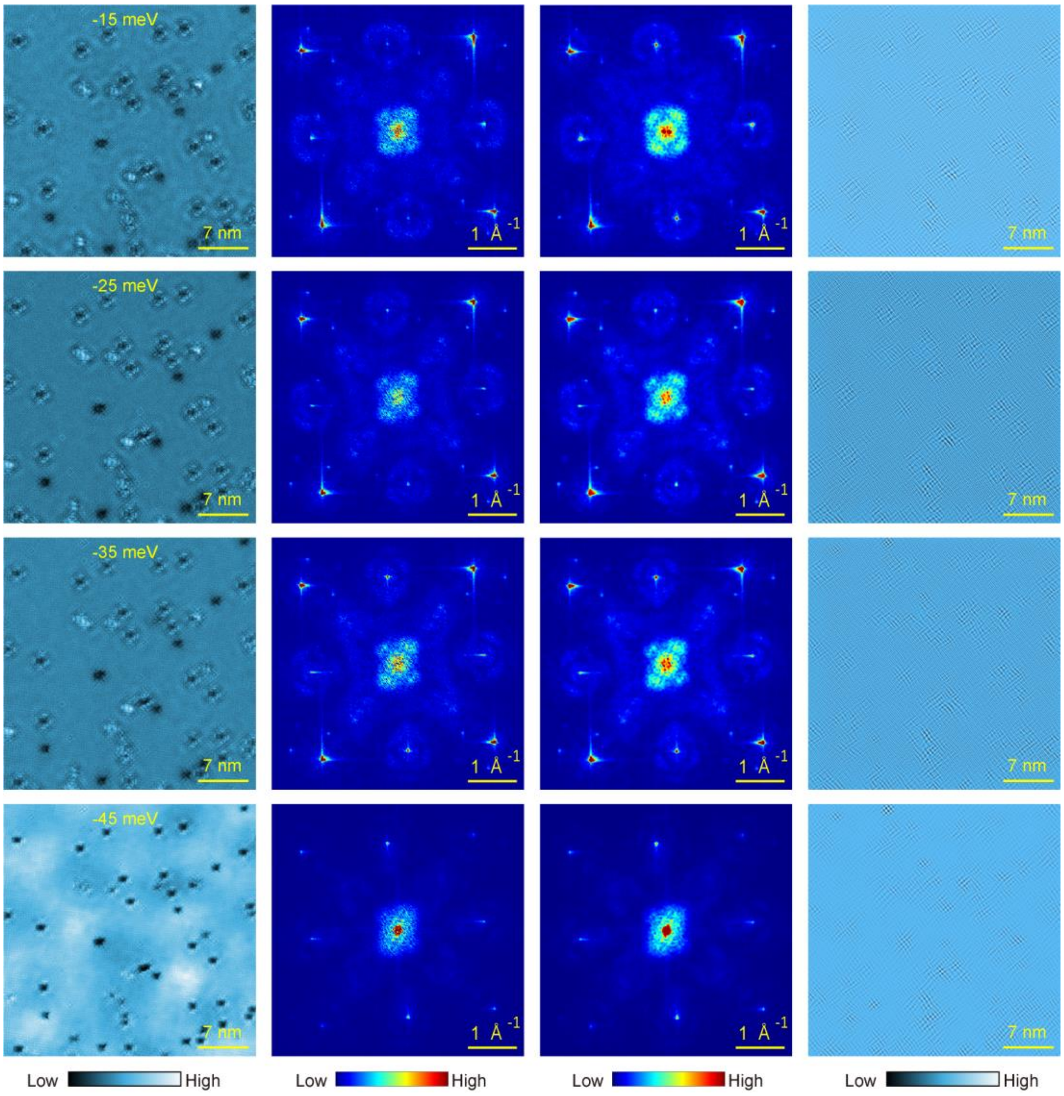

Fig. S4 Additional datasets of dI/dV maps collected in the $Fe_{1-x}Se$ (x = 0.54%) region shown in Fig. 1a. The corresponding raw FFT images, smoothed FFT images, and the iFFT images with FFT intensities around $\mathbf{q}_{Fe}$ and $\mathbf{q}_{2Fe}$ considered are also listed.

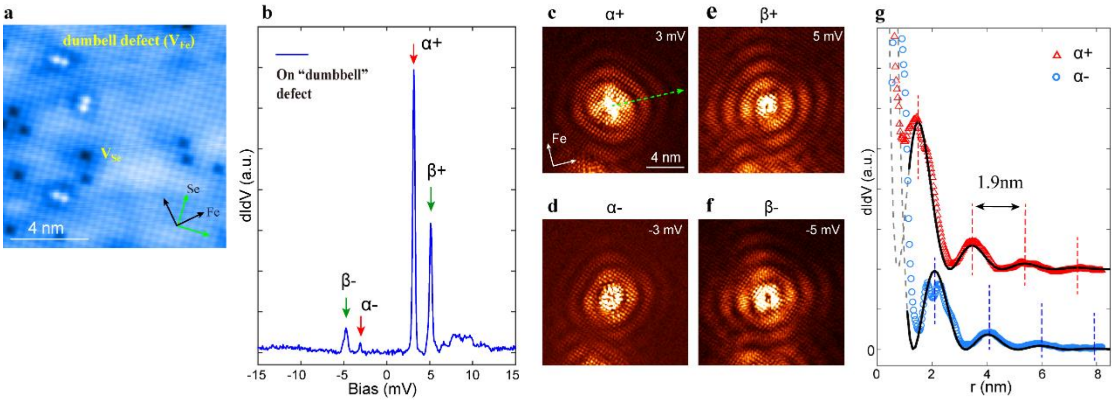

Fig. S5 Measurement on the spatial distribution of the in-gap YSR states. a Topographic image of the FeSe surface (V_b = 160 meV, I_t =10 pA). **b** Normalized dI/dV spectra taken on a type-I Fe-site defect (V_b = 15 meV, I_t = 60 pA, ΔV = 0.3 mV). Two pairs of sharp in-gap states are observed. **c-f** dI/dV maps around a type-I Fe-site defect, taken at energies of α±, β± states. **g** dI/dV line profiles of α± states along

the Fe-Fe direction (green arrow in **c**). This figure is cited from another of our manuscripts that are under review as well.

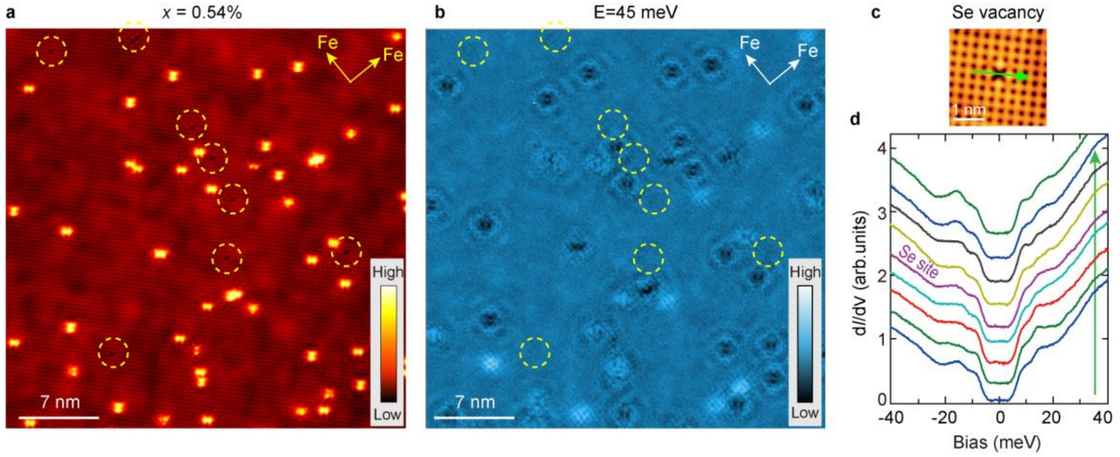

Fig. S6 Effect of Se vacancies on superconductivity. a,b STM image and corresponding dI/dV map at E=45 meV for the $Fe_{1-x}Se$ region with x = 0.54%. Se vacancies are marked out by the yellow dashed circles. **c** Topographic image of an Se vacancy. **d** Series of dI/dV spectra taken along the green arrow shown in **c**. Se vacancies have no influence on superconductivity of $Fe_{1-x}Se$ plane, and do not pin checkerboard pattern.

5. Additional datasets for the extended checkerboard patterns observed in several $Fe_{1-x}Se$ regions with various x values

The extended checkerboard pattern has been observed in several $Fe_{1-x}Se$ regions when $x > 1.8\%$, as shown in Figs. S7-S13.

Figure S7 shows additional datasets of the dI/dV maps collected in the $Fe_{1-x}Se$ (x = 2.2%) region shown in Fig. 3a. The corresponding raw FFT images, smoothed FFT images and the iFFT images with FFT intensities around $\mathbf{q}_{Fe}$ and $\mathbf{q}_{2Fe}$ considered are listed as well. The extended checkerboard pattern is obvious in the dI/dV maps at positive energies, while they are not so intuitive at negative energies due to the influence of dominating Se lattice, as illustrated by the enhanced intensity of $\mathbf{q}_{Se}$ Bragg peak in the FFT image under E = -40 meV. All the FFT images are basically C_4-symmetric. By performing the iFFT of FFT features around $\mathbf{q}_{Fe}$ and $\mathbf{q}_{2Fe}$ to remove the interference of QPI signals and Se lattice, the extended checkerboard pattern is more distinguishable in both positive and negative energies. Moreover, L-map, obtained by calculating (dI/dV)/(I/V) at each point to correct the current deviation, is another method to effectively eliminate the influence of Se lattice. As a result, the charge modulations at negative energies can be clearly seen, as shown in Fig. S8.

Figure S9, S10, S12 present the datasets measured in $Fe_{1-x}Se$ regions with x = 1.8%, 3.2% and 6.35%, respectively, where the extended checkerboard pattern is observed as well. Figure S11 and S13 show the L-maps of the data for x = 3.2% and 6.35%, where the charge modulations at negative energies can be clearly resolved.

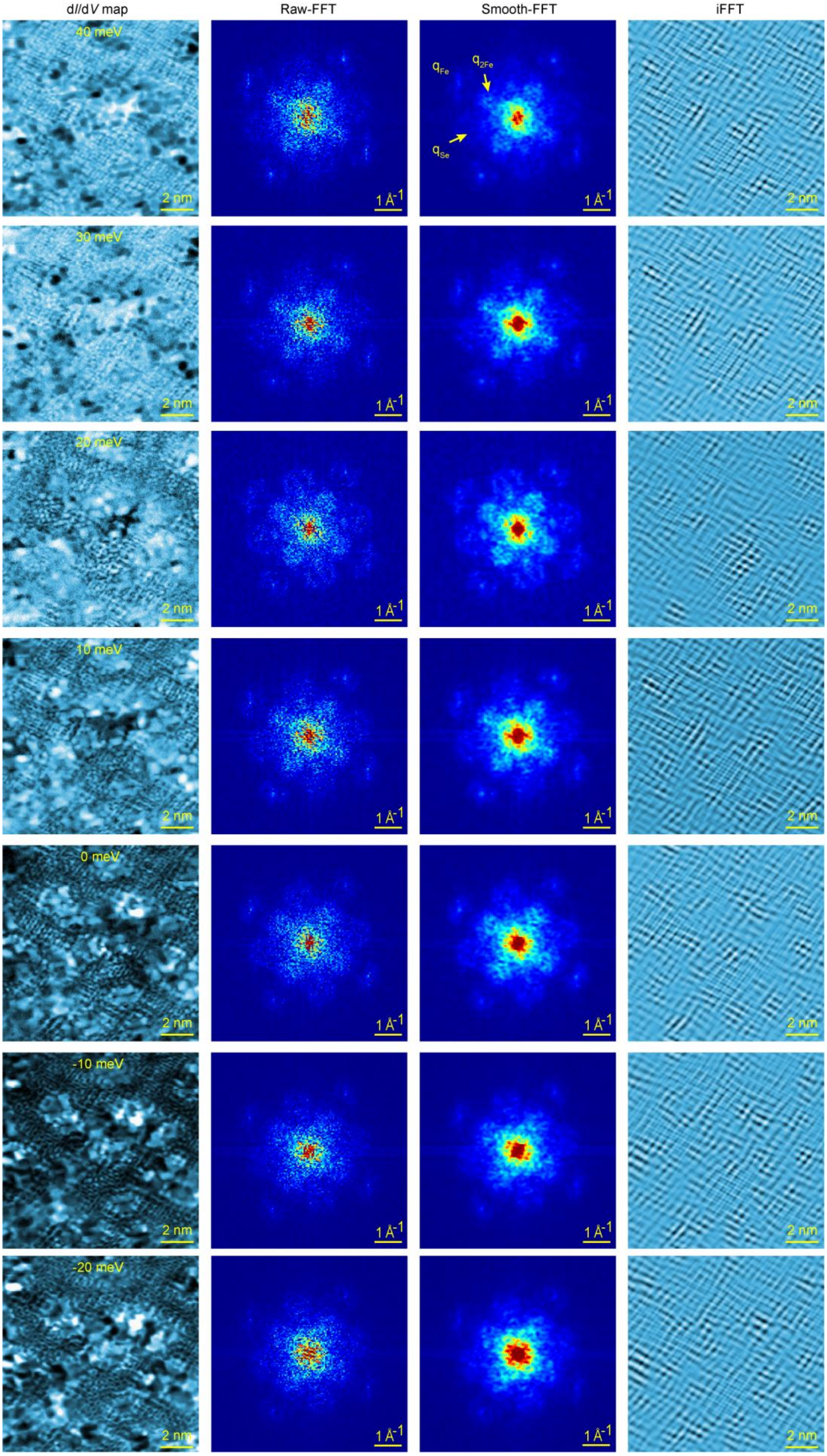

dI/dV map
Raw-FFT
Smooth-FFT
iFFT
40 meV
30 meV
20 meV
10 meV
0 meV
-10 meV
-20 meV
q_Fe
q_2Fe
q_Se
2 nm
1 Å^-1

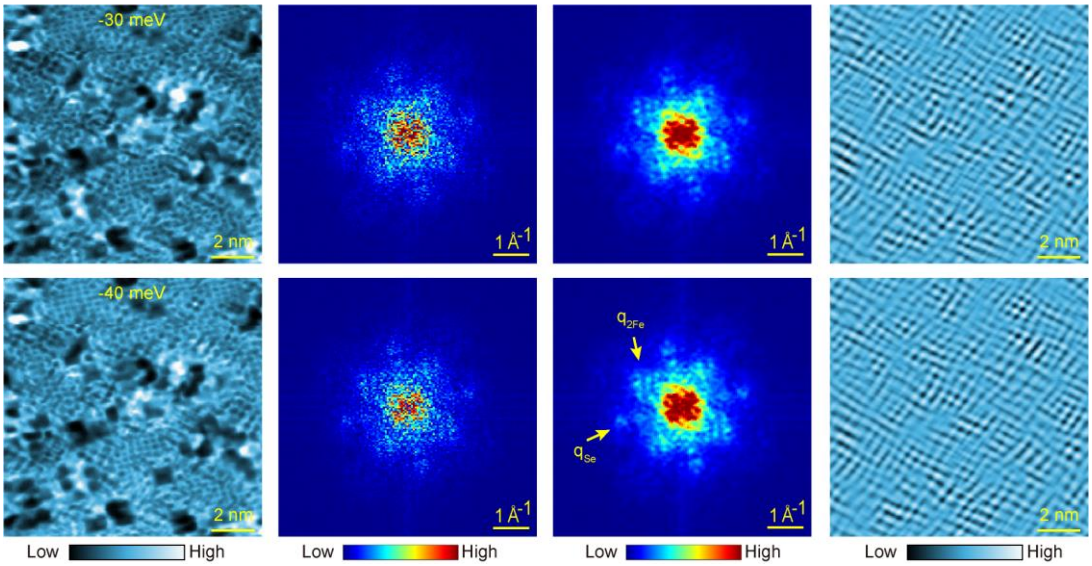

Fig. S7 Additional datasets of d*I*/d*V* maps collected in the $Fe_{1\text{-}x}Se$ (*x* = 2.2%) region shown in Fig. 3a. The corresponding raw FFT images, smoothed FFT images, and the iFFT images with FFT intensities around $\mathbf{q}_{Fe}$ and $\mathbf{q}_{2Fe}$ considered are also listed.

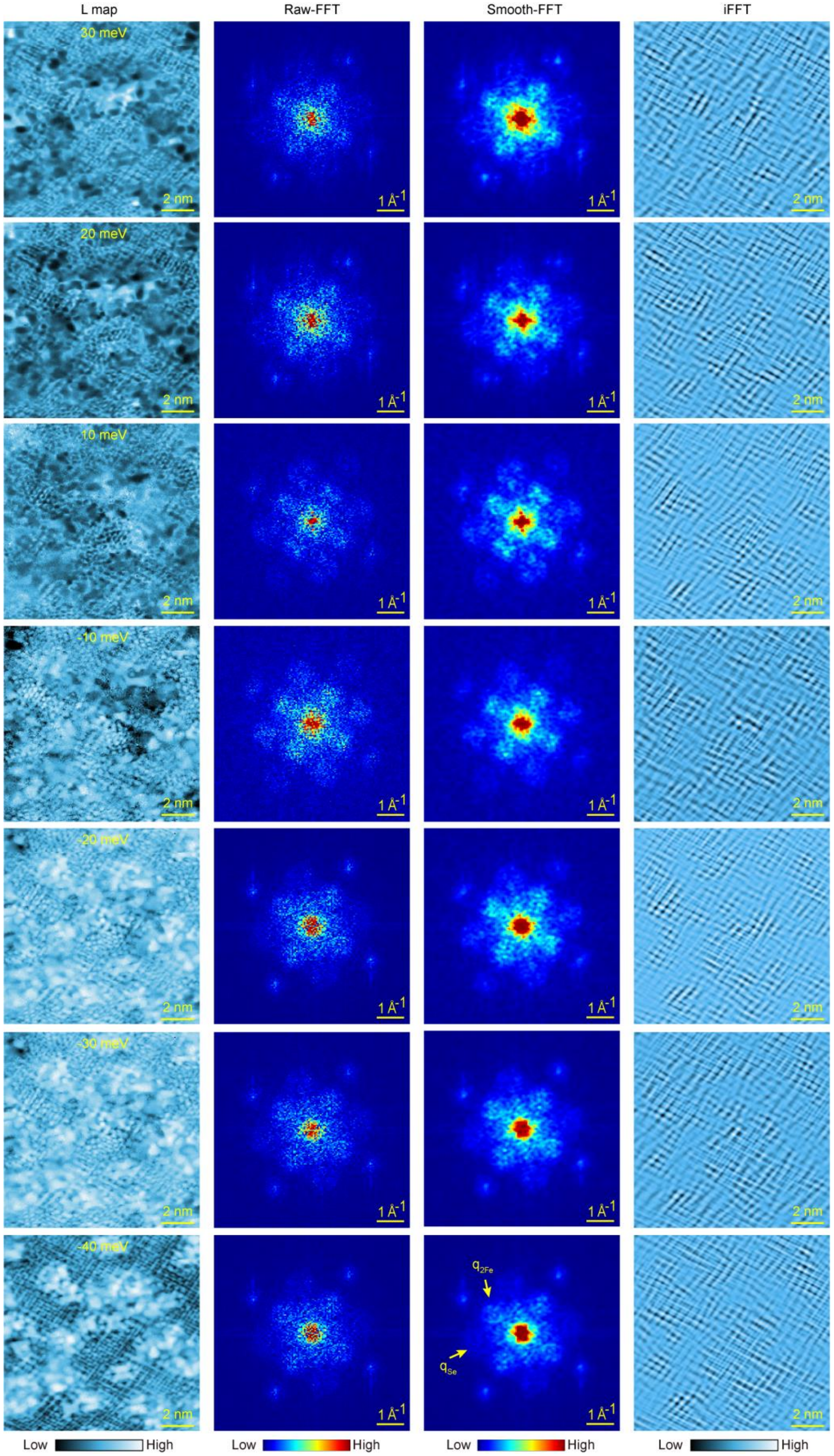

Fig. S8 L-maps for the data shown in Fig. S7, together with the corresponding raw FFT images, smoothed FFT images, and the iFFT images with FFT intensities around $\mathbf{q}_{Fe}$ and $\mathbf{q}_{2Fe}$ considered.

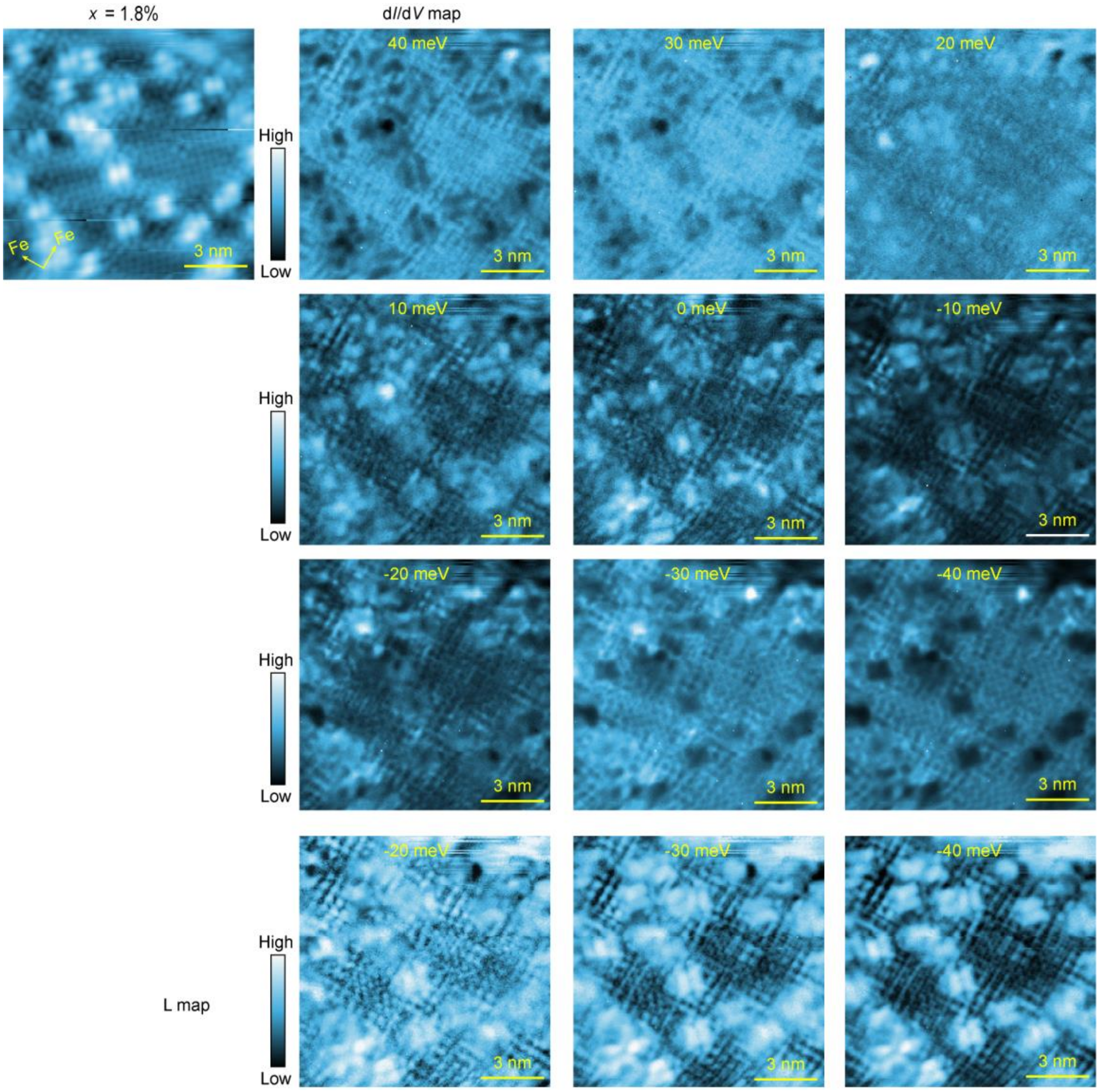

Fig. S9 STM image and corresponding d*I*/d*V* maps taken in an $Fe_{1-x}Se$ region with x = 1.8%. L-maps for the negative energies are also listed. The extended checkerboard pattern is obvious in the d*I*/d*V* and L-maps.

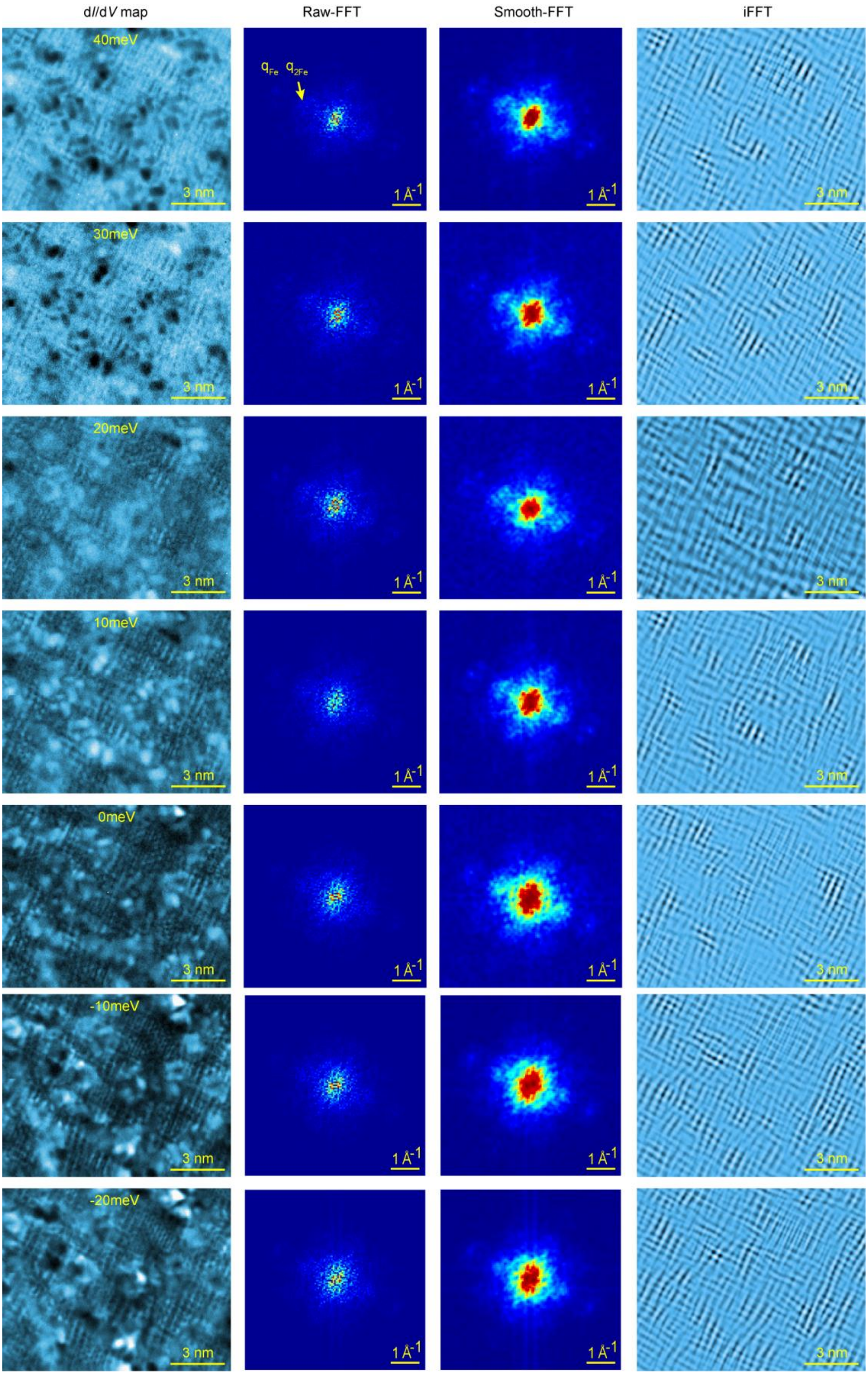

dI/dV map
Raw-FFT
Smooth-FFT
iFFT
40meV
30meV
20meV
10meV
0meV
-10meV
-20meV
q_Fe q_2Fe
3 nm
1 Å^-1

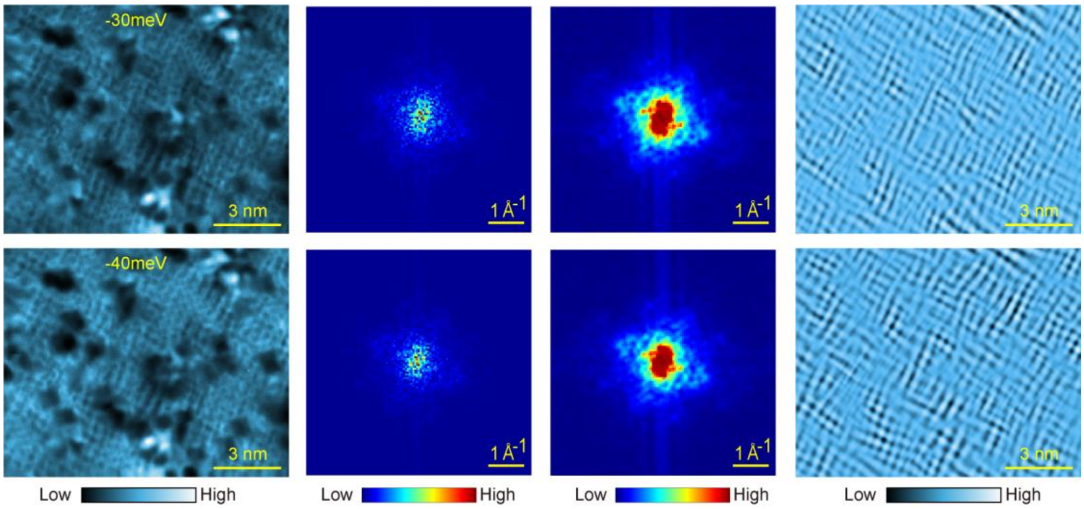

Fig. S10 Additional datasets of d*I*/d*V* maps collected in the $Fe_{1-x}Se$ (*x* = 3.2%) region shown in Fig. 4a. The corresponding FFT images, smoothed FFT images, and the iFFT images with $\mathbf{q}_{Fe}$ and $\mathbf{q}_{2Fe}$ intensities considered are listed here.

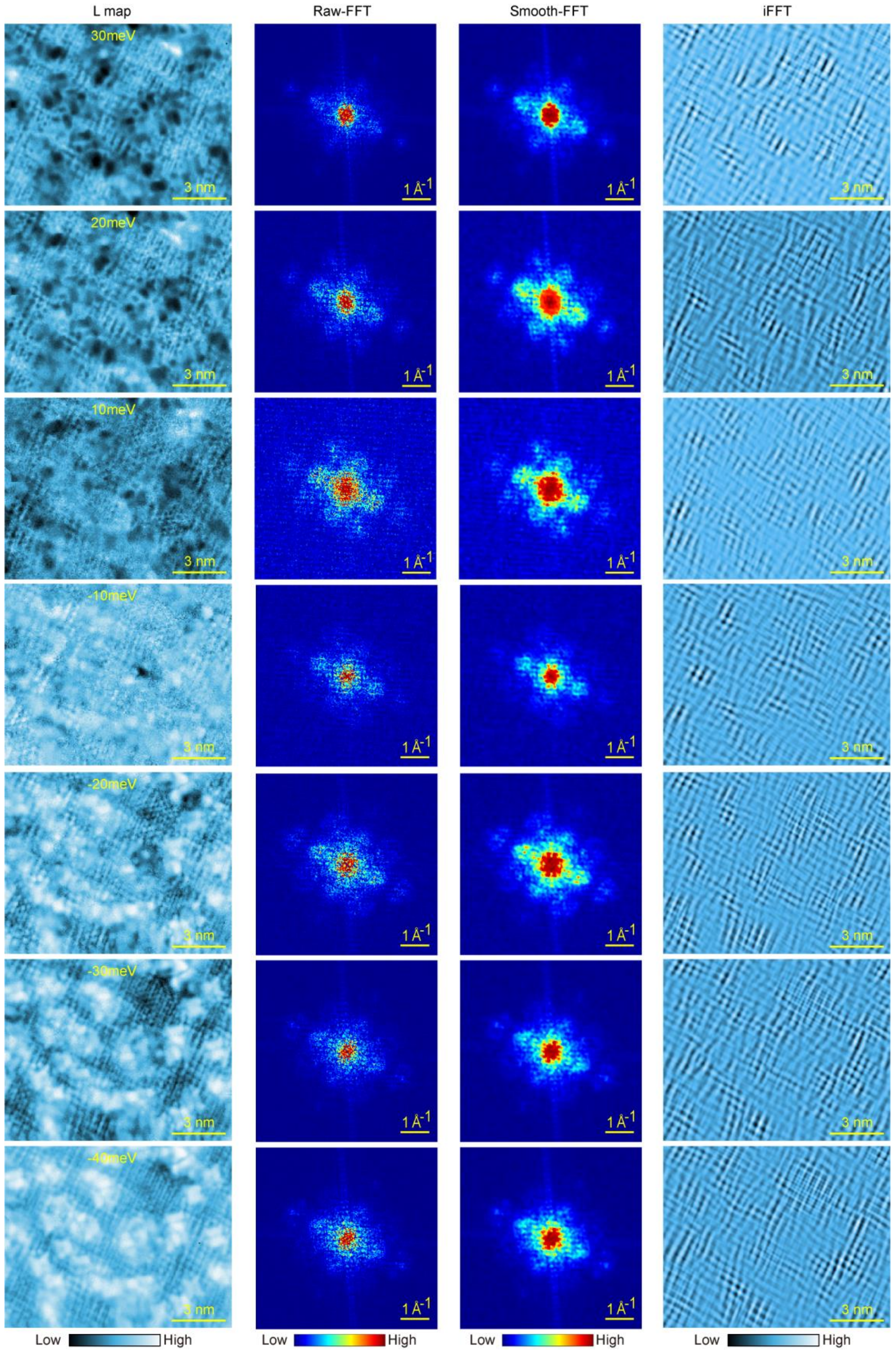

Fig. S11 L-maps for the data shown in Fig. S10. The corresponding raw FFT images, smoothed FFT images, and the iFFT images with FFT intensities around $\mathbf{q}_{Fe}$ and $\mathbf{q}_{2Fe}$ considered are also listed.

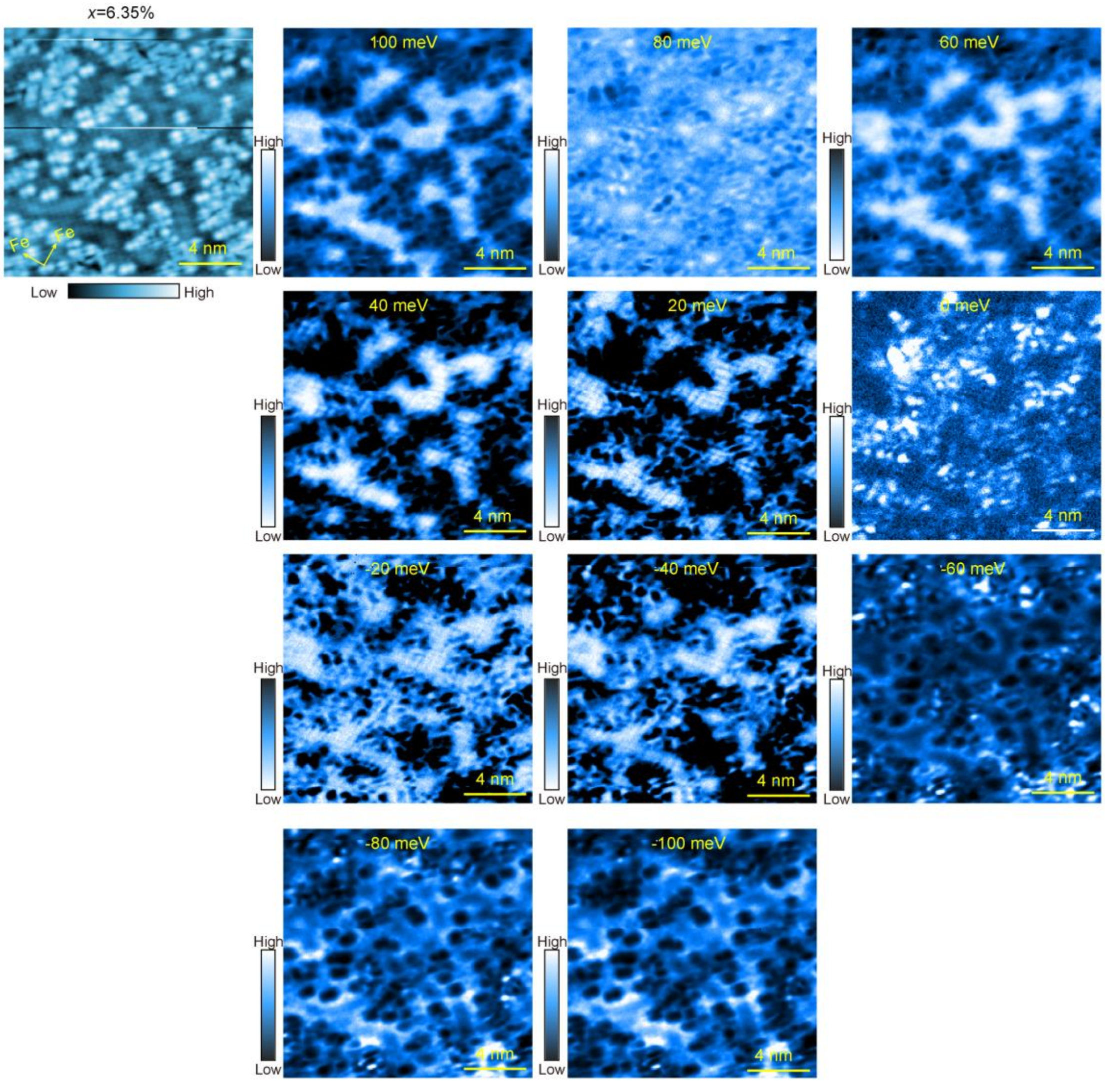

Fig. S12 Additional datasets of d*I*/d*V* maps collected in the $Fe_{1-x}Se$ (*x* = 6.35%) region shown in Fig. 4e.

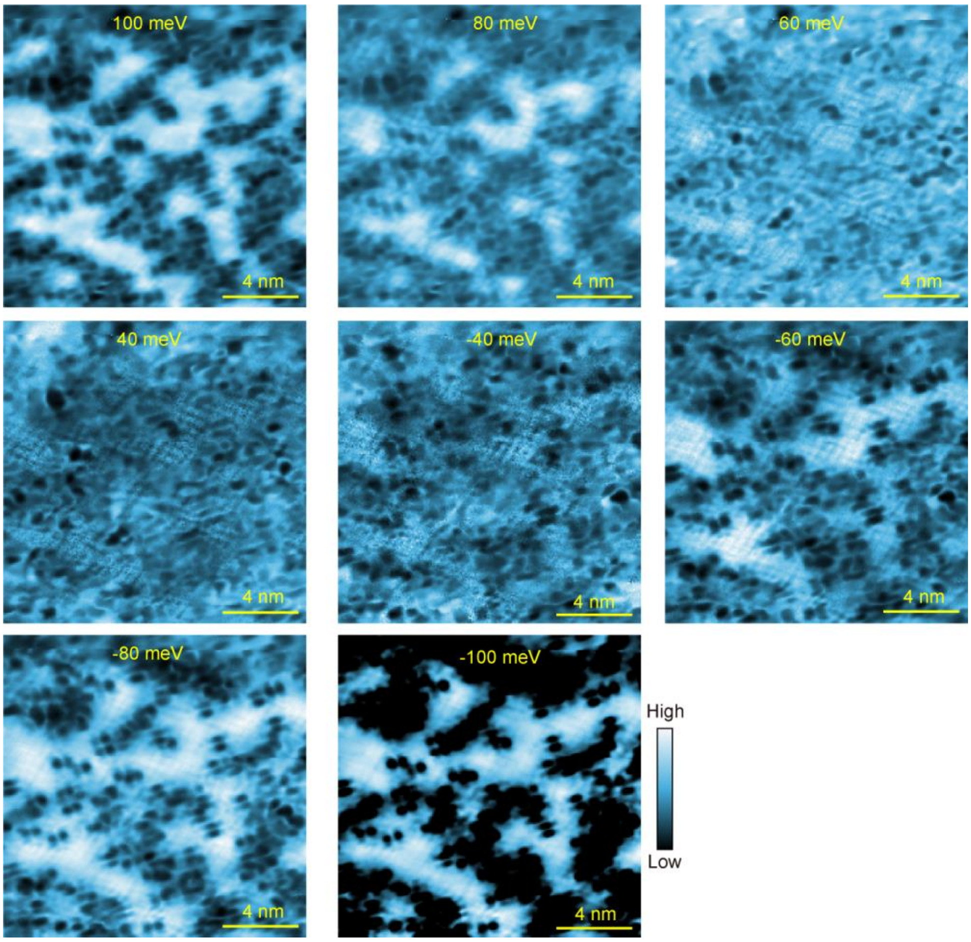

Fig. S13 L-maps for the data shown in Fig. S12. The checkerboard pattern can be seen clearer here.

6. Appearance of unidirectional charge stripes in the vicinity of Fe-site defects

In our study, we find that the checkerboard charge order dominates in the whole phase space of Fe-site defect tuned $(Li_{0.84}Fe_{0.16}OH)Fe_{1-x}Se$, while the unidirectional charge stripes were occasionally observed in the vicinity of Fe-site defects, as shown in Fig. S14. For x=2.2% shown in Fig. S14a,b, there are two regions showing rather unidirectional charge stripes, as indicated by the yellow boxes, while the other regions remain the C_4-symmetric checkerboard patterns. The directions of the charge stripes in these two regions are perpendicular, excluding the tip effect. Moreover, the unidirectional charge stripes are more obvious in some regions (indicated by yellow boxes) for x=3.2% shown in Fig. S14c,d, while it's not so obvious in the other regions. The charge stripe is not evident for $Fe_{1-x}Se$ region with x=6.35%, as shown in Fig. S12 and S13. For the unidirectional charge stripes, it seems likely that a $2a_{Fe}$-period modulation in a small local area is preserved along one of the Fe-Fe lattice directions, but is smeared in the perpendicular direction, as displayed in Fig. S14e,f and sketched in Fig. S14g.

Moreover, we find that the appearance of unidirectional charge stripes strongly depends on the local environment. Roughly, it's more likely to appear in the vicinity where Fe-site defects gathered together, as shown in Fig. S14a,c; we suspect that the gathered Fe-site defects in a very small sample region may locally distort the lattice and induce a large local strain that breaks the C_4-symmetry locally, which needs further investigation.

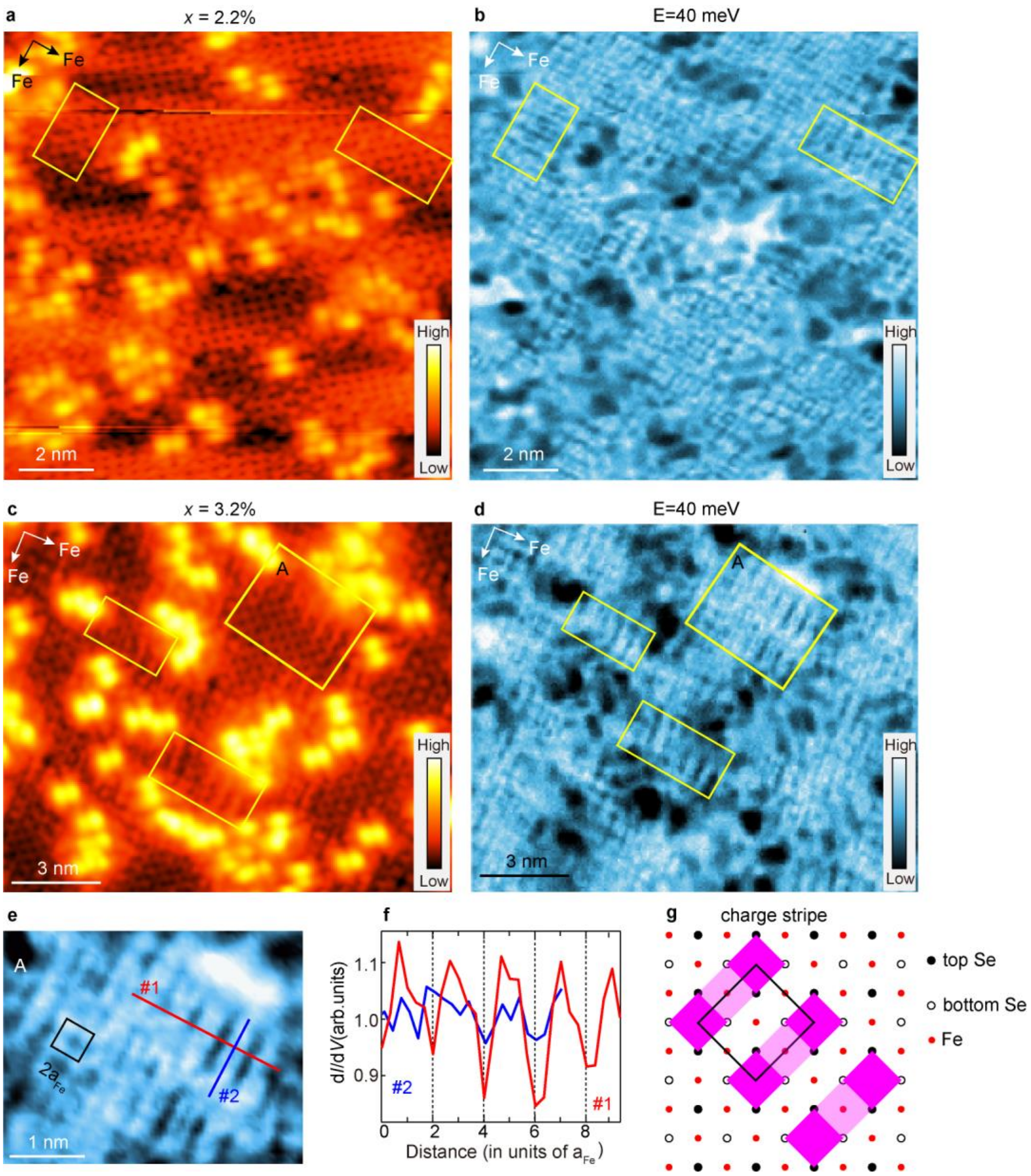

Fig. S14 Appearance of local unidirectional charge stripes. Atomically resolved topographic images (**a**,**c**) for $Fe_{1-x}Se$ regions with x = 2.2% and 3.2% and corresponding dI/dV maps at E=40 meV (**b**,**d**). The yellow boxes indicate the regions of unidirectional charge stripes. **e** The enlarged image of area A marked by the yellow box in **d**. The black box shows the unit cell of the charge order with a $2a_{Fe}$ period. **f** Spatial LDOS profiles taken along cuts #1 and #2 in **e**. **g** Schematic illustration of the fine structure of charge stripes atop the Fe and Se lattice. Unit cell of this structure is marked by the black box. The magenta squares represent higher LDOS observed by STM, the dark and light colors show difference in LDOS intensity.

7. Calculation of the in-plane correlation lengths of charge orders observed in different $Fe_{1-x}Se$ regions with varying x values

From the dI/dV maps shown in part 5 of this text, we can see directly that the observed charge order is short-ranged. Here we try to calculate the in-plane correlation lengths of the short-ranged charge orders in different $Fe_{1-x}Se$ regions with varying x. The first column in Fig. S15 show the dI/dV maps used for calculations, and the second column in Fig. S15 present the filtered iFFT images

considering only the broad $\mathbf{q}_{2Fe}$ spots. A standard 2D autocorrelation of these images is carried out and symmetrized along the high-symmetry directions, which gives the third column of Fig. S15. To obtain the in-plane correlation length ξ of the short-range charge order, we extract the line profiles along the black dashed lines in the third column of Fig. S15 and fit them with a standard function as mentioned in previous report[3]. The function has a form of $A(x) = A_0 e^{(-\frac{x}{\xi})} \cos(k_{CDW} x) + B_0$, and since k_{CDW} is known, and B_0 is the average of the random noise in the autocorrelation image ($B_0 << 1$), we fit the curve with a form of $A_0 e^{(-\frac{x}{\xi})}$. The result is shown by the blue dashed curves in the fourth column of Fig. S15, and the fitted ξ values are all close to $4a_{Fe} \sim 5a_{Fe}$.

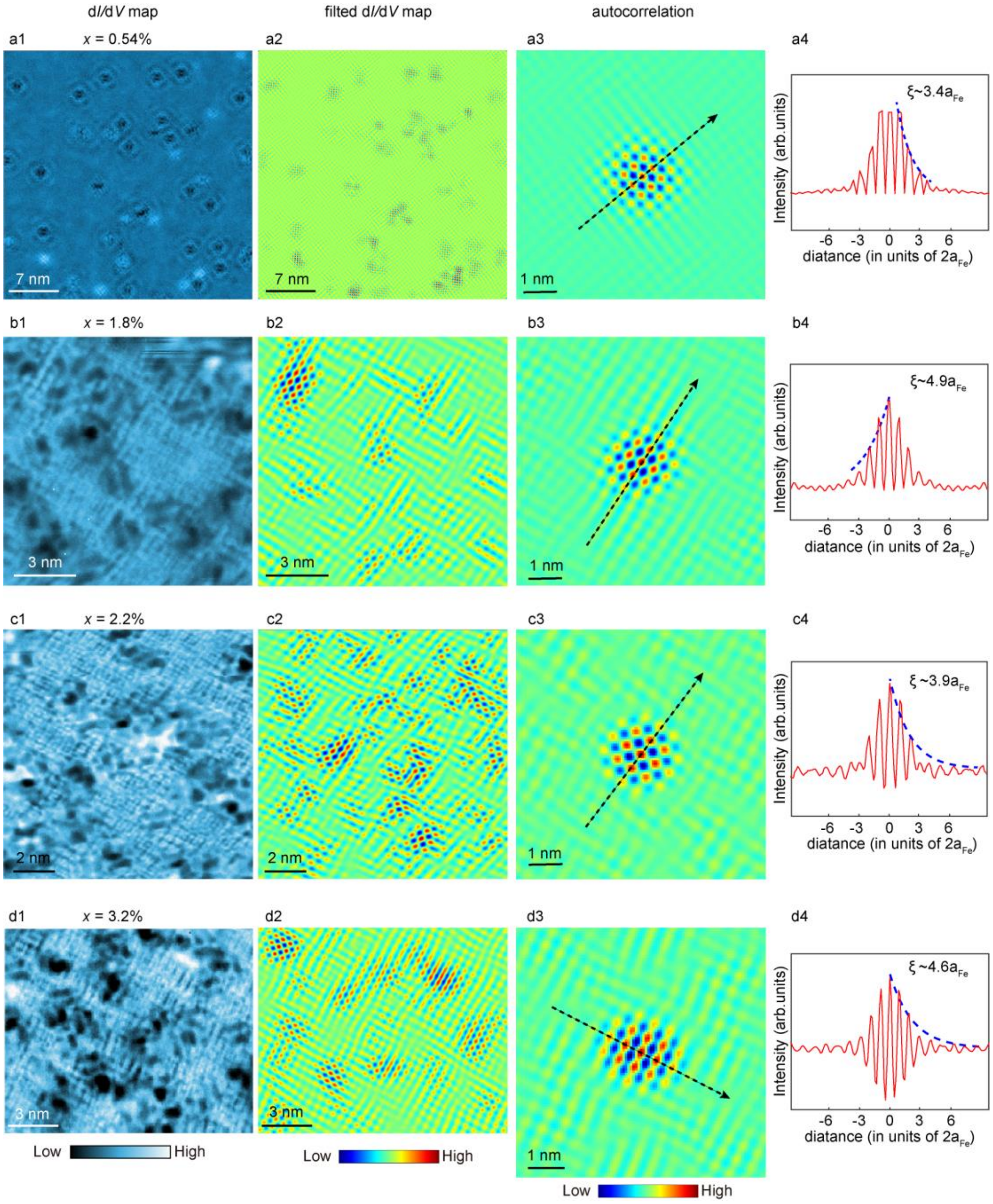

Fig. S15 Calculation of in-plane correlation lengths of short-range charge orders observed in

different $Fe_{1-x}Se$ regions with varying x values. The first column shows the dI/dV maps for different $Fe_{1-x}Se$ regions, and their filted images with only broadened $\mathbf{q}_{2Fe}$ spots considered are shown in the second column. The third column are the corresponding autocorrelation of the images shown in the second column. The fourth column show the line profile across the black dashed lines shown in the third column and the fitted curves (blue dashed curves).

8. Simulated real space spin and charge patterns based on inelastic neutron scattering measurements

As discussed in the main text, INS measurements on optimally doped $(Li_{0.84}Fe_{0.16}OD)Fe_{1-x}Se$ have found low energy magnetic excitations centered around four momenta ($\pi \pm \delta\pi$, π) and (π, $\pi \pm \delta\pi$) (δ~0.38), as shown in Fig. 5a. Such a momentum distribution is broad, covering commensurate values such as $\delta = 0.5$. Here we qualitatively simulate the resulting spin density and corresponding charge distributions with various $\mathbf{Q}_i$ vectors shown in Fig. 5a by assuming $\delta = 0.5$, the simulation method has been discussed in the main text. Figs. S16a1-n1 show the combinatorial configurations of $\mathbf{Q}_i$ vectors for the simulation, and the simulated spin/charge patterns are plotted in Figs. S16a2-n2 and S16a3-n3, respectively.

For Figs. S16a1-d1, only one single $\mathbf{Q}_i$ is considered, the resulting single-**Q** SDWs and corresponding charge orders are shown in Figs. S16a2-d2 and S16a3-d3, all display tilted stripes with respect to the Fe-Fe lattice, which differ greatly from the experimental patterns. Then we consider a multiple-**Q** SDW picture, which is achieved by the coherent addition of multiple SDWs with varying $\mathbf{Q}_i$ $(\mathbf{Q}_1, \mathbf{Q}_2, \mathbf{Q}_3, \mathbf{Q}_4, \mathbf{Q}'_1, \mathbf{Q}'_2, \mathbf{Q}'_3, \mathbf{Q}'_4)$. Figs. S16e1-h1 show several combinatorial configurations of double $\mathbf{Q}_i$ vectors, and the resulting double-**Q** SDWs and corresponding charge orders are plotted in Figs. S16e2-h2 and S16e3-h3, respectively. For Figs. S16e3-g3, the charge patterns cannot reproduce the experiments in terms of the period, the direction and the detailed intra-unit-cell structures. For Fig. S16h3, the combination of $\mathbf{Q}_4$ and $\mathbf{Q}'_4$ seems to produce a charge pattern with the same period and orientation to the experiments.

Moreover, considering the complete magnetic excitation patterns in Fig. 5a, it is more natural to select the four horizontal or vertical $\mathbf{Q}_i$ vectors for simulation (Figs. S16i1,j1). The resulting quadruple-**Q** SDWs are shown in Figs. S16i2,j2 and both have a $4a_{Fe} \times 2a_{Fe}$ unit cell; the corresponding charge orders in Figs. S16i3,j3 show the horizontal or vertical stripes with a $2a_{Fe} \times a_{Fe}$ unit cell. Furthermore, the coherent addition of the total eight $\mathbf{Q}_i$ vectors (Fig. S16k1) results in a SDW with a $4a_{Fe} \times 4a_{Fe}$ unit cell (Fig. S16k2) but a charge pattern with a $2\sqrt{2}a_{Fe} \times 2\sqrt{2}a_{Fe}$ unit cell that rotated 45° with respect to the Fe-Fe lattice (Fig. S16k3). This is inconsistent with the experimentally observed checkerboard charge pattern. Instead, the incoherence overlap of the vertical and horizontal stripes shown in Figs. S16i3,j3 could qualitatively reproduce the checkerboard pattern (Fig. S16l3). In Fig. S16m1, when the intensity of the horizontal SDW is 1/3 of that of the vertical SDW, the incoherent overlap of these two SDWs results in a C_2-symmetric charge order with a $2a_{Fe} \times 2a_{Fe}$ unit cell, as shown in Fig. S16m3, which can qualitatively reproduce the experimentally observed local unidirectional charge stripes. Besides, in Fig. S16n1-S16n3, we show that the incoherent overlap of the horizontal or vertical charge stripes induced by two double-**Q** SDWs can also give a checkerboard charge pattern.

In summary, after comparing the above simulated patterns with experimental results, among all possible multiple-**Q** pictures shown here, we think the charge patterns resulted from the incoherent addition of the horizontal and vertical double-**Q** or quadruple-**Q** SDWs, as shown in Figs.

S16n3 and S16l3, fit the experimental checkerboard pattern well. How to distinguish these two configurations and which one fits the experiments better require further theoretical investigations. Moreover, the intensity variance between the horizontal and vertical SDWs results in the observed local charge stripes, suggesting a possible origin of the unidirectional charge stripes.

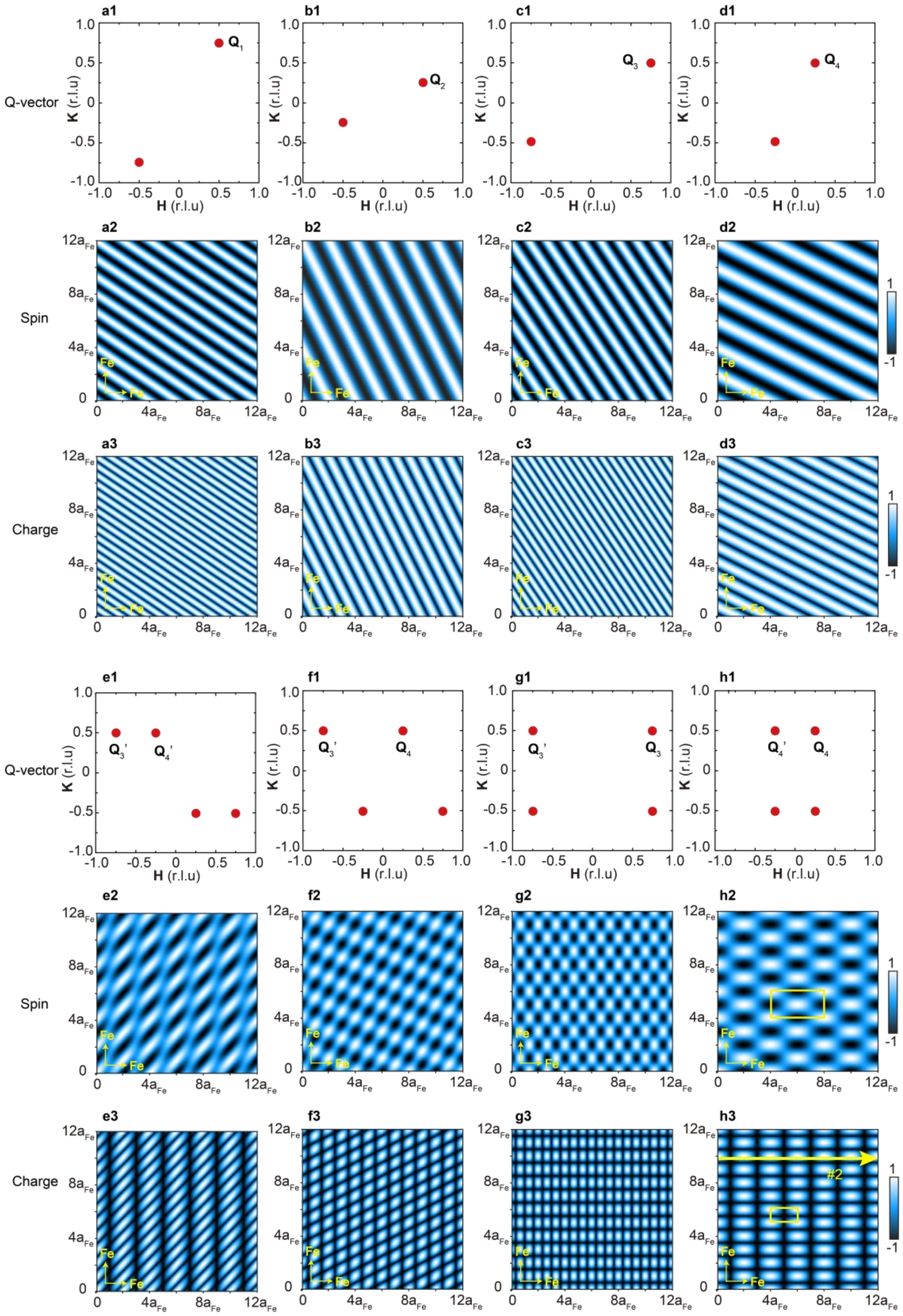

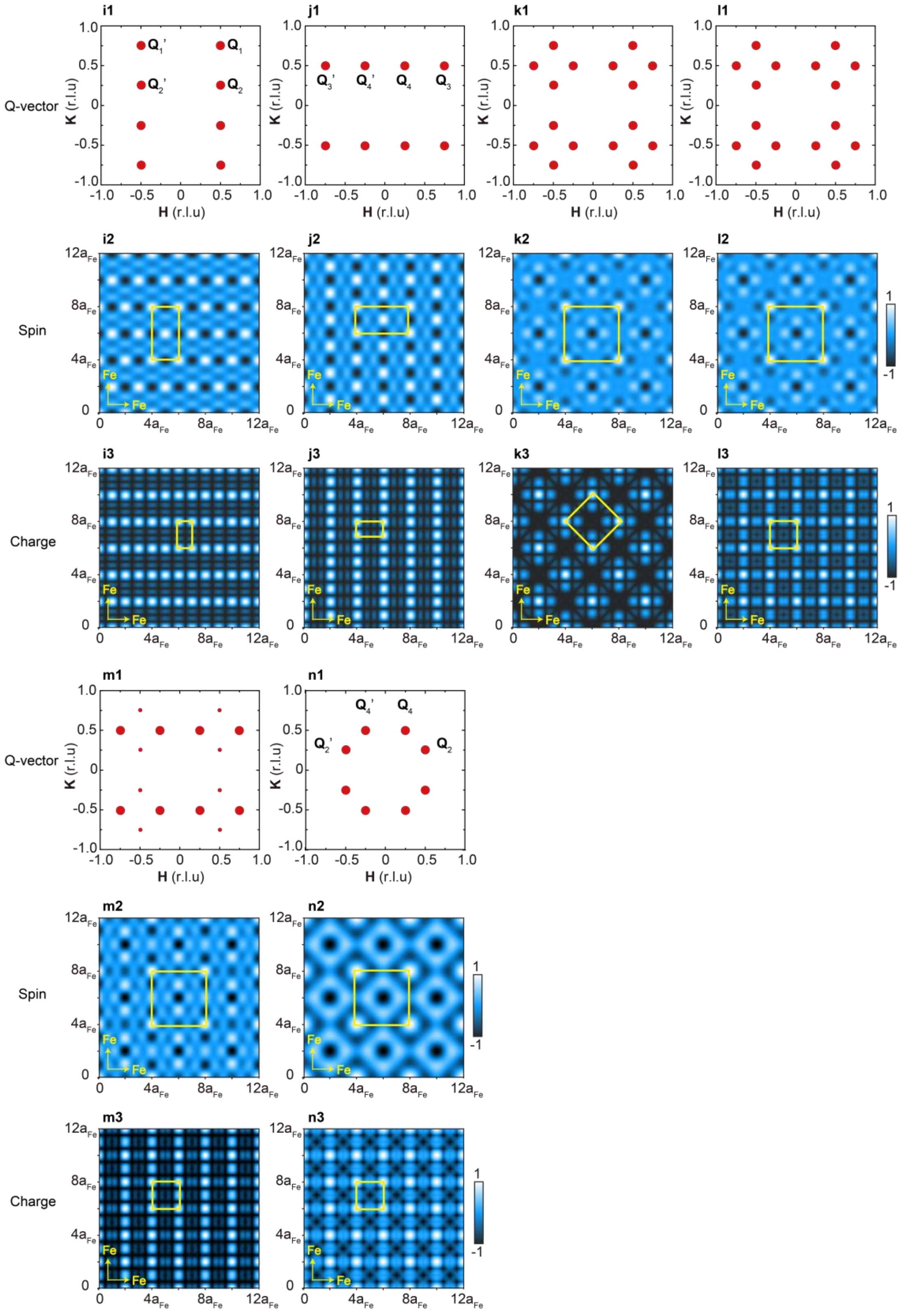

Fig. S16 Simulation of spin and charge patterns in real space. a1-n1 Momentum distributions of the combinatorial **Q**-vector configurations for the simulation. The red dots mark the commensurate spin fluctuation momenta, while the smaller red circles in **m1** suggest that the intensity of these **Q**-vectors is a third as strong as the other **Q**-vectors. **a2-n2** Simulated spin patterns. The multiple SDWs with varying

$\mathbf{Q}_i$ vectors are coherently added. **a3-n3** Corresponding charge patterns. Most of the charge patterns are obtained directly by the coherently added SDW patterns except those shown in **l3-n3**, where the charge patterns are obtained by the incoherent overlap of the charge patterns driven by two vertical and horizontal SDWs considering the combination of $(\mathbf{Q}_1, \mathbf{Q}_2, \mathbf{Q}_1', \mathbf{Q}_2')$ and $(\mathbf{Q}_3, \mathbf{Q}_4, \mathbf{Q}_3', \mathbf{Q}_4')$ for **l3 and m3**, and the combination of $(\mathbf{Q}_2, \mathbf{Q}_2')$ and $(\mathbf{Q}_4, \mathbf{Q}_4')$ for **n3**, respectively.

Supplementary References:

1. Sun, H. *et al.* Soft chemical control of superconductivity in lithium iron selenide hydroxides $Li_{1-x}Fe_x(OH)Fe_{1-y}Se$. *Inorg. Chem.* **54**, 1958 (2015).

2. Huang, Y. L. *et al.* Matrix-assisted fabrication and exotic charge mobility of (Li,Fe)OHFeSe superconductor films. Preprint at https://arxiv.org/abs/1711.02920 (2017).

3. C. J. Arguello, S. P. Chockalingam, E. P. Rosenthal, L. Zhao, C. Gutierrez, J. H. Kang, W. C. Chung, R. M. Fernandes, S. Jia, A. J. Millis, R. J. Cava, and A. N. Pasupathy, Visualizing the charge density wave transition in $2H\text{-}NbSe_2$ in real space, *Phys. Rev. B* **89**, 235115 (2014).
